# Effectiveness of the Therapeutic Community Model in Addiction Treatment: A Retrospective Pilot Study in French Prisons

**DOI:** 10.3390/healthcare11111523

**Published:** 2023-05-23

**Authors:** Théodore Vinais, Aurélie Lacroix, Thibaut Gelle, Philippe Nubukpo

**Affiliations:** 1 Pôle Universitaire de Psychiatrie d’Adulte de la Personne Agée et d’Addictologie (PUP3A), Centre Hospitalier Esquirol, 87000 Limoges, France; 2 Unité de Recherche et d’Innovation, Centre Hospitalier Esquirol, 87025 Limoges, France; 3 Inserm U1094, IRD U270, EpiMaCT—Epidémiologie des Maladies Chroniques en Zone Tropicale, Institut d’Epidémiologie et de Neurologie Tropicale, OmegaHealth, University of Limoges, CHU Limoges, 87042 Limoges, France

**Keywords:** addictive disorder, prison, therapeutic community

## Abstract

Background: In France, addiction care in prison usually consists of nurses’ interventions, medical care and socio-educational programs, but new alternatives have arisen, namely the therapeutic community (TC) model. This pilot study aims to evaluate the effectiveness of this prison-based TC in comparison with classic and socio-educational care offered in French prisons. Methods: To compare these three types of prison-based care, two detention centers’ files were screened for use of multiple drugs, willingness to participate and absence of psychiatric comorbidities incompatible with group therapy. A custom questionnaire was built based on the fifth version of the Addiction Severity Index. It investigates medical status, employment and support, primary addiction status, legal status, social/familial status and psychiatric status through various items. Results: Our sample only consisted of male repeat offenders with a mean age of 37.7 ± (9.1) years. Primary addiction status improvement was observed for all care studied but was more important in TC than in classic care. Self-esteem and social/familial status saw significant improvement throughout TC care. Conclusions: The TC model represents an alternative to classic and socio-educational care in French prisons. More studies are needed to assess the extent of the benefits provided on both the medical side and economic side.

## 1. Introduction

Drug use is often associated with higher crime rates [1,2]. In France, studies estimate that 20% of inmates were convicted for crimes concerning illicit drug possession, use or sale [3,4]. Furthermore, between 60% and 80% of inmates report using drugs in the weeks prior to incarceration [4,5,6]. 

Even in prison, there are possibilities for substance use disorder treatment. In 1994, France inscribed in its laws that inmates should have access to the same standard of care as any other patient and shifted responsibility for prisoners’ health from the Justice Ministry to the Health Ministry.

Since then, usual care is performed by “Sanitary Units in Penitentiary Settings” (classic care). Those units are mostly composed of nursing and medical staff, but in order to deal with drug use care, help is enlisted from psychiatric hospitals and specialized institutions [7]. Doctors specialized in addictology and educators are then tasked to help ensure that prisoners benefit from the same standard of care as they would outside of prison. 

Since 2015, an alternative has been offered to inmates wanting to work on their social skills and drug use during incarceration. This socio-educational program, inspired by Spanish prisons, is called “Respect Module” (Respect). This program puts an emphasis on rehabilitation through work. Inmates sign a contract describing their objectives and their commitment to achieving them. By doing so, they agree to follow a well-defined code of conduct that also grants them more leeway during their personal time (e.g., easier access to phones, parlors, gyms, etc.). They are then tasked to choose a personal activity plan (group work on videos, music, drawing, yoga, building a resume for work, etc.) and to respect a specific schedule throughout the day [8].

Finally, in 2017, a third type of care was introduced as an experimentation unit. The Rehabilitation Unit for Drug Users (RUDU) was created to try and apply the therapeutic community (TC) approach to drug use care in prison. The TC model consists of medium- to long-term (one year or more) accommodation structures that do not solely focus on addiction pathology but aim to rehabilitate patients so they can behave and live accordingly with social rules. To that end, patients are held accountable and are expected to become positive and reliable actors of the community. This “community as a method” approach [9] values one’s personal involvement in the group dynamic in order to change oneself. This way, the individual will improve himself by appealing to the group’s resources through the development of his own social skills [10]. Staff mainly play a role in promoting interactions among peers through educational activities and monitoring residents’ and groups’ statuses [11]. TC’s effectiveness is already well described overseas in regular settings for its effect on drug use both during TC and after discharge [12,13]. These effects mainly consist of a reduction in drug episodes that can be reduced by 77% 3 months after discharge and sustained for a longer period. Prison-based TC programs also have an effect on drug use, notably on relapse [14,15], which can decrease by 15%. This effect on relapse can in turn diminish the likelihood of recidivism from 17% up to 39%, mainly by reducing drug-related offenses [15,16,17]. Prison-based TC was also shown to be effective in reducing rearrests [16], even if it has only been described as a small to moderate effect when aftercare is not involved after release.

In RUDU, prisoners’ days are organized with multiple group activities focused on resolving issues using communication skills, learning how to express their feelings when speaking with their peers or using arts and creations to do so. Expression groups help redefine their relation with the products of their addiction as well as their central role in the prisoners’ problems. 

This unit was implanted in the Neuvic detention center thanks to the collaboration of penitentiary, associative and medical actors. RUDU is mostly composed of specialized educators as well as social workers, an addictologist, a psychologist, probation officers and even prison guards. RUDU staff often have interviews with prisoners to assess their progress, review recently encountered problems or talk about their near-term prospects (i.e., preparing for a leave of absence or the first days of life after release).

The TC model differs vastly from classic care in French prisons. The TC model focuses and relies on social rehabilitation to improve the overall state of the inmate, while classic care solely aims to work on the addiction pathology through medical interviews. The TC model gives structure to the inmates’ stay in prison by encouraging them to participate in regular group activities and discussions. It is also important to note that the TC model allows for closer monitoring with regular interviews than classic care.

This pilot study aimed to evaluate care based on the TC model in French prisons through the RUDU experimental unit and compare its effectiveness with other types of care available in French prisons such as classic care and socio-educational programs. To that end, a questionnaire was purposely built based on the fifth version of the Addiction Severity Index (ASI) [18,19]. The primary objective was to evaluate the evolution of the overall addictive disorder, focused on the primary substance used or to be given up by the resident. The secondary objectives were to evaluate and compare effects on other factors linked with delinquency and addiction such as social interactions, self-esteem and reincarceration.

## 2. Methods

### 2.1. Study Design

This retrospective study was conducted by the Esquirol Hospital Center of Limoges. This study was funded by the Regional Health Agency of the Nouvelle-Aquitaine region of France and was registered in the Health Data Hub (health-data-hub.fr, accessed on 1 April 2023). A letter was sent to the known addresses of all inmates whose files were to be included to inform them about the study and give them the opportunity to voice their opposition towards the use of their data.

### 2.2. File Selection

The study took place in two prisons in the Nouvelle-Aquitaine region of France. The first one, offering the three different types of care, was the Neuvic detention center. The second one was the Mont-de-Marsan penitentiary center, which offers both classic and socio-educational care for drug users. Socio-educational files were only selected in Mont-de-Marsan. TC files were only selected in Neuvic. Classic care files were selected in both centers.

Files were selected if the inmate had spent at least 3 months in the studied unit’s care and were meeting RUDU ‘s admission criteria, listed as follows: drug user seeking help and treatment, use of multiple substances and absence of heavy psychiatric comorbidities that would prevent group therapy. Some additional pairing criteria were required for the selection of files from other types of care in order to ensure comparability and prevent bias. The files were paired according to the duration of care, primary substance of the addiction pathology, age group and psychiatric comorbidities. 

### 2.3. Study Procedures

The study started by including all adequate TC files from the 103 files available for the 2017 to 2020 period. Only 39 TC files were adequate and therefore included. These 39 files were used to build the pairing grid based on the criteria described above. Classic care and socio-educational files were then selected according to the pairing grid. For those, the end of treatment was arbitrarily defined to be similar to the original TC files they were to be paired with (data beyond the end of treatment defined were not studied). In total, 23 classic care files and 13 socio-educational care files were included for a total of 75 files. A total of 16 of the 23 classic care files were from the Neuvic detention center, while 7 were from the Mont-de-Marsan detention center.

After all the files were selected, a questionnaire based on the 5th version of the Addiction Severity Index (ASI) [18,19,20] was filled out using the files as sources of information. The ASI is a hetero-questionnaire that takes interest in two periods: the lifetime and the last 30 days. It could not be used directly since it requires a dialogue with the patient to fill in the data corresponding to the last 30-day period. Therefore, our questionnaire was built by removing the family history domain from the ASI and replacing the two periods of interest for severity grades (the lifetime and the last 30 days) with the initiation of care studied and the end of said care. The questions relating to the last 30 days and self-evaluation were also removed from all the remaining domains. Severity grades were given to all the domains evaluated by our questionnaire, namely: medical status, employment and support, primary addiction status, legal status, social/familial status and psychiatric status. Grades were not reflective of a single questionnaire item but rather represented the global status of an explored domain. For example, primary addiction status incorporated items regarding abstinence, reduction in consumption, ability to feel, measure and voice craving, ability to ask for help, ability to refuse when the substance was offered by another inmate, compliance with treatment, etc. Similarly, psychiatric status was composed of psychiatric history, diagnosis, treatment for psychiatric or emotional problems, etc. Finally, along the same lines, employment and support status incorporated items regarding previous employment, education level and diploma or lack thereof, main source of income, training during incarceration, etc. Grades ranged from 0 to 9, with 0 being the lowest and describing the absence of problem, while 9 was the highest and described an extreme problem needing emergency care and treatment.

Grades were given jointly after a full examination of all the files of each inmate by both investigators; one was a medical doctor specialized in addiction medicine and psychiatry who focused on medical status, primary addiction status and psychiatric status domains of our questionnaire, and the other was graduate in addiction medicine with a master’s degree in biology who focused on employment and support, legal status and social/familial status of our questionnaire. This second investigator also selected and revised the files.

Self-esteem scores presented in this study were self-reported and ranged from 1 to 5, with 5 being the highest. In this study, abstinence took into consideration the willingness aspect of undertaking a period without consumption as a step of recovery and not simply because the substance could not be procured. The duration of abstinence was measured in days. Multiple periods of abstinence were not taken into account (i.e., the longest one was favored). Abstinence data were centered on the main substance of choice for each inmate. The data were only taken into account if measured during in-prison care. Reincarceration rates were measured 90 and 180 days after release from the detention during which the studied care occurred.

### 2.4. Statistical Analysis

This study questionnaire provided both qualitative and quantitative data. Quantitative variables were described using means and standard deviation, and qualitative variables were expressed in numbers and percentages. To analyze this study’s qualitative data, Chi^2^ tests were used. If the sample size was deemed too small, Fisher’s test was used. When paired (i.e., the same population but multiple measurements of a criterion at different times during care), quantitative data were analyzed using Wilcoxon’s test. Otherwise, Mann–Whitney’s test was used. All statistical analyses were conducted on IBM SPSS Statistics version 26 (IBM Corp., Armonk, NY, USA) with a statistically significant threshold set at 0.05.

## 3. Results

### 3.1. Study Population

The study population age ranged from 20 years old to 59 years old (Mean (M) = 37.7 ± 9.1 years). The mean duration of care was 175.8 ± 53.7 days. All inmates were male.

In total, 79.5% (N = 31) of the TC population had benefited from care previously, while only 39.1% (N = 9) from the classic care and 46.2% (N = 6) from the socio-educational care population had (Table 1). 

TC and classic care were compared in populations’ prior access to substance use care (Fisher, *p* = 0.018). No significant difference was revealed when comparing TC and socio-educational care (Fisher, *p* = 0.057) (Table 1).

### 3.2. Global Effectiveness of Care Regarding Addictive Disorder

Evolutions of severity grades were used to compare the effectiveness of care. Admission scores corresponded to the severity grade awarded from the first interview with a professional involved in care. Similarly, the latest score was given according to the file’s last interview, usually within a week of release. Delta represents the progress made during care. The higher the Delta value is, the more progress was made during care.

When looking at primary addiction status, the comparability of the admission score (Table 2) ensured that the baseline was similar among the three types of care studied. The baseline showed tremendous problems for all the files included (M = 8.76 ± 0.59). The Delta values indicated that progress was made across all types of care studied (M = 2.6 ± 0.82). When comparing TC’s and classic care’s Delta, we found that TC’s effect was significantly superior (Mann–Whitney, *p* = 0.026) (Table 2). However, the same could not be said when comparing TC and socio-educational care (Mann–Whitney, *p* = 0.862). This indicated that the global progress in the addiction pathology is higher with TC than with classic care but not higher than with socio-educational care.

### 3.3. Comparing Care Effectiveness in Improvement of Psychiatric Status and Financial Situation

For both psychiatric status and employment/support, the baselines indicated important issues requiring care for all populations studied (M = 7.95 ± 1.15 and M = 7.93 ± 1.6, respectively). Similarly to primary addiction status, progress was achieved in both psychiatric status and employment/support across all three types of care studied (M = 2.49 ± 0.79 and M = 2.31 ± 1.7, respectively). No significant difference was detected when comparing TC to the two other types of care studied (Table 2 and Table 3).

### 3.4. Possible Factors of Improvement Promoted by the Therapeutic Community Model

Both TC and socio-educational care shared the same rate of abstinence at 84.62%. The classic care abstinence rate was at 69.57% and was not significantly different from TC (Fisher, *p* = 0.348). TC had the longest mean duration of abstinence (M = 199.81 ± 137.05 days) followed by classic care (M = 166.25 ± 95.84 days). Socio-educational care had the shortest mean duration of abstinence (M = 142.18 ± 62.55 days). No differences were observed when comparing TC’s duration of abstinence with the other populations (Mann–Whitney, p_classic care_ = 0.392, p_socio-educational_ = 0.241).

Self-reported self-esteem and social/familial status could only be found in TC’s proprietary files, so we only took interest in progress being made during TC. When comparing admission scores to latest scores for both of these items (Table 4), the significant improvement made during TC was revealed.

### 3.5. Effects on Reincarceration Rates

Finally, the reincarceration rates at 90 and 180 days after release were analyzed. The data could only be obtained for the former Neuvic detention center prisoners (39 in TC and 16 in classic care), while for both, the reincarceration rates were lower at 90 days after release (TC = 10.26%; classic care = 7.69%) than 180 days after release (TC = 25%; classic care = 18.75%), and no significant difference was found between the two types of care studied (Fisher, 90 days *p* = 0.212; 180 days *p* = 0.342).

## 4. Discussion

This study gives us the first data on the TC model’s effectiveness on drug use disorder in French prisons. While this study did not allow us to establish profiles of prisoners most likely to respond to a particular type of care, we can still identify disparities between their populations. Indeed, our study showed that TC’s population is more likely to have previously accessed professional help in substance use care than the population undergoing classic care. This could indicate that prisoners that have already encountered classic care in addiction, whether it was in prison or not, tend to be motivated to take part in more intensive type of care such as the one the TC model provides [9,16].

Our results point towards the TC model’s superiority in its capacity to induce better improvement overall in addiction status when compared to classic care. These findings are consistent with previous studies in Europe and North America [21]. This result can be explained by the more intensive nature of TC, although it remains difficult to identify which components are the most beneficial [15]. Socio-educational care is also more intensive than classic care and relies on personal involvement, which could explain why the overall improvement in addiction status is similar for socio-educational care and TC.

When comparing other aspects of care such as psychiatric status and employment/support, we were not able to detect differences between the three units. The lack of significant improvement in psychiatric status is consistent with the limited effect on mental health in the TC literature [22]. This means that the TC model could benefit from stronger psychotherapy components in order to broaden the range of its benefits to inmates.

The addiction pathology and its treatment are complex and involve multiple factors. Self-esteem is described as one of these factors, as lower self-esteem is correlated to more substance use [23,24]. The TC model allows residents to work on this personality trait as it is also linked with group membership and belongingness [25]. De Leon suggests that group membership can constitute a replacement for the bond with the object of addiction [9]. Belongingness is also known to be a crucial part of TC used in order to promote change and in turn improve self-esteem [26]. Therefore, TC’s positive effects on self-esteem could be linked to belongingness, and both of them might play an important role on the overall progress made by the inmates on their addictive disorder. In that sense, they could constitute a secondary index of this criteria of effectiveness. Familial/social status is also an important factor in addiction status improvement that is worked on with the TC model used in RUDU [27]. In this unit, prisoners learn to improve their communication skills in expression groups with the help of other residents, care professionals and even prison guards.

Our findings indicate no significant differences, neither in terms of promoting attempts of abstinence nor in the duration of such endeavors. Our results were unexpected, as TC’s positive effects on relapse have been described [14] and could be expected to translate into a positive effect on abstinence. However, abstinence is not the main purpose of the TC model because the recovery process and goals are not solely based on the addictive disorder but instead on a “whole person” approach, which could explain the absence of a significant effect on this criterion [9]. Still, it could be a meaningful indicator for some prisoners’ progress with their addictive disorder that should not be dismissed. It also means that future studies should strongly consider other criteria to measure addiction status, such as craving frequency and intensity or reductions in drugs consumed both in nature and quantity, in addition to abstinence.

Reincarceration rates were of particular interest, as prison-based TC has shown effectiveness in reducing reincarceration [14] and recidivism [28,29]. We were not able to detect any significant differences in our study, probably due to the low number of files containing data on this subject. Indeed, data could only be obtained for 39 TC files and 16 files for classic care. The short time period with up-to-date information (i.e., up to 180 days after release) could also have played a role in those results. Studying reincarceration rates for a longer period after release could allow a significant difference to emerge. Other items could also be studied, such as recidivism, which was considered by O. Mitchell and colleagues as the most reliable and comparable outcome criterion [15,17].

Finally, RUDU allows for care professionals to systematically search for adequate aftercare in the couple of weeks leading to release. It is well described that aftercare is one of the most important components for substance use treatment success and for preventing relapse, recidivism [1,16,17] and reincarceration [14,30,31]. Similarly, the study period being focused on in-prison care and therefore not allowing us to measure the progress retained after (whether still in prison or released) could have deprived us of meaningful indicators of effectiveness [32]. Hence, the TC model represented by RUDU might offer much more societal advantages and rehabilitation capabilities than our study is able to reveal.

### Limitations

This retrospective study encountered two main limitations while comparing the different options of addiction care in French prisons. Firstly, the files’ content is highly dependent, not only on the detention center, but also on the specific type of care received by the prisoner. This disparity in files’ content and the lack of standardized tools for evaluation could have hindered the comparison by misrepresenting both the progress and the difficulties encountered by prisoners and fields teams alike. Secondly, a robust cost analysis of the three types of care studied is also missing from this study. Although we originally aimed to include a cost-effectiveness analysis, we were prevented from producing such an analysis by the lack of available data and the poor comparability of those obtained. Despite these shortcomings, the economic data retrieved could help design a more robust analysis that could be used in a future prospective study. Such a prospective study should be focused on inmates’ fates after release from the three different programs studied here. Follow-up would be crucial to assess how much of the progress, both in the addiction pathology and in social rehabilitation, made in prison is retained when inmates are no longer in a secure environment. Particular attention should be given to aftercare and whether or not inmates return to a known problematic social environment or rather choose to start anew to avoid the repetition of criminal behavior. Finally, the long-term economic impact should be assessed to help policy makers decide how to help develop and fund such care programs.

## 5. Conclusions

This study represents a first look into the TC model in French prisons through the special unit that is RUDU. Although TC’s effectiveness has already been demonstrated in reducing substance use and criminality in conventional settings and in prisons overseas, such data are lacking in France [21]. This study points towards stronger improvement in the addiction pathology granted by the TC model than by classic care in prison. It also shows that the TC model is not solely focused on the addiction pathology but rather on social rehabilitation and can significantly improve other characteristics such as social/familial status and self-esteem. More studies are required on the subject, particularly when it comes to follow up and the fates of inmates after release. To that end, it is important to implement standardized tools to monitor, evaluate and improve such treatments, as TC’s more intensive care can be more beneficial for some inmates than the classic procedure available. The clear identification of the most efficient components of the TC model and the profiles of prisoners most likely to stay in care and take advantage of it is crucial to ensure its efficiency [9,15,21,25,28]. It could also be interesting to evaluate the benefits of RUDU and the TC model on an economic scale to see if it is compatible with healthcare and the justice system in France. To that end, cost–benefit analyses relying on standardized tools such as ASI or the Drug Abuse Treatment Cost Analysis Program (DATCAP) [33,34] could provide interesting insights to both field teams and policy makers.

## Figures and Tables

**Table 1:** Study population characteristics (N = 75).

Demographics	N	Percent
Age categories		
18–29 years old	12	16%
30–39 years old	34	45.30%
40–49 years old	18	24%
50 years old and older	11	14.70%
Type of care		
TC	39	52%
Classic care	23	30.70%
Socio-educational care	13	17.30%
Primary substance of addiction		
Alcohol	32	42.70%
Opiates	24	32%
Cannabis	7	9.30%
Cocaine	12	16%
Previous care/treatment in addictology		
Yes	46	61.30%
No	10	13.30%
Unknown	19	25.30%

**Table 2:** Evolution of health-related severity grades during care (N = 75).

		TC (N = 39)	Classic Care (N = 23)	Socio-Educational Care (N = 13)
		Mean ± SD	Mean ± SD	Mean ± SD
Primary addiction status	Admission score	8.62 ± 0.71	8.96 ± 0.21	8.85 ± 0.55
	Latest score	5.85 ± 0.71	6.65 ± 0.88	6.23 ± 0.93
	Delta	2.77 ± 0.71	2.30 * ± 0.82	2.62 ± 1.04
Psychiatric status	Admission score	7.97 ± 1.14	8.13 ± 1.10	7.54 ± 1.27
	Latest score	5.31 ± 1.28	5.87 ± 1.46	5.15 ± 1.34
	Delta	2.67 ± 0.81	2.26 ± 0.75	2.38 ± 0.77

Test (Mann–Whitney) compares TC’s Delta to Delta from other study populations. * *p* < 0.05.

**Table 3:** Evolution of employment and support severity grades during care (N = 70) *.

	TC (N = 39)	Classic Care (N = 20)	Socio-Educational Care (N = 11)
	Mean ± SD	Mean ± SD	Mean ± SD
Admission score	7.69 ± 1.87	8.10 ± 1.33	8.45 ± 0.82
Latest score	5.33 ± 1.74	5.90 ± 1.83	6.09 ± 1.76
Delta	2.36 ± 1.18	2.20 ± 1.36	2.36 ± 1.50

* Three files from classic care and two files from socio-educational care did not contain enough data to be graded.

**Table 4:** Effectiveness of TC on social/familial status and self-esteem improvement.

	Admission Score	Latest Score	
	Mean ± SD	Mean ± SD	*p* (Wilcoxon)
Social/familial status (N = 39)	7.33 ± 1.59	5.15 ± 1.6	<0.001
Self-esteem (N = 31)	3.26 ± 0.86	3.65 ± 0.84	0.028

## Data Availability

The data presented in this study are available on request from the corresponding author. The data are not publicly available because it is derived from a sensitive and protected sample of the population.
